# Persistent Immune Thrombocytopenia as a Post‐Acute Sequelae of COVID‐19: A Case Report

**DOI:** 10.1002/puh2.70412

**Published:** 2026-10-06

**Authors:** Riazullah, Hakim Ullah Wazir, Aafeen Mujeeb, Hamid Ahmad, Hameed Ur Rehman, Inibehe Ime Okon

**Affiliations:** ^1^ Department of Medicine Medical Teaching Institute—Mardan Medical Complex Mardan Pakistan; ^2^ Department of Medicine Medical Teaching Institute—Lady Reading Hospital Peshawar Pakistan; ^3^ Department of Medicine Khyber Medical College Peshawar Pakistan; ^4^ Department of General Internal Medicine Sligo University Hospital Sligo Ireland; ^5^ Department of Research Medical Research Circle (MedReC) Bukavu Democratic Republic of the Congo

**Keywords:** coronavirus disease 2019 (COVID‐19), immune thrombocytopenia (ITP), long‐term complications, persistent thrombocytopenia, post‐acute sequelae

## Abstract

Immune thrombocytopenia (ITP) is a recognized hematologic complication associated with coronavirus disease 2019 (COVID‐19), but its persistence years after acute infection remains uncommon. We present a case of a 52‐year‐old male who was diagnosed with COVID‐19 in June 2020. Following recovery from the acute illness, he had persistent fatigue, reduced energy levels, and poor appetite. Laboratory testing at his first follow‐up revealed thrombocytopenia with a platelet count of 60,000/mm^3^. He had no bleeding manifestations or organomegaly. A trial of oral corticosteroids in a tapering dose produced a partial response, with the platelet count rising to 94,000/mm^3^. Subsequently, a bone marrow biopsy was performed, confirming the diagnosis of ITP, excluding infiltration and dysplasia. Over the following 5 years, the patient continued to demonstrate persistent thrombocytopenia requiring a close clinical monitoring and follow‐up. This case highlights persistent ITP as a long‐term sequela of COVID‐19, extending more than 4 years after the initial infection. It underscores the importance of considering post‐COVID hematologic complications in patients presenting with chronic thrombocytopenia and emphasizes the need for ongoing surveillance and individualized management strategies.

## 1 Introduction

Immune thrombocytopenia (ITP) is an autoimmune condition defined by isolated platelet counts below 100,000/mm^3^ without another clear cause [1]. Patients can present without symptoms or with bleeding that ranges from mild mucocutaneous spots to severe hemorrhage. The disorder can occur on its own or as a secondary problem in viral infections like human immunodeficiency virus (HIV), EBV, CMV, and HCV. A few reports have also linked it to severe acute respiratory syndrome coronavirus 2 (SARS‐CoV‐2) infection [2].

Low platelet counts are common in coronavirus disease 2019 (COVID‐19) and appear in about one‐third of patients [3]. Several pathways may drive this finding. SARS‐CoV‐2 can affect blood forming cells and megakaryocytes. Systemic inflammation can alter the bone marrow microenvironment and impair normal platelet production. Liver involvement can reduce thrombopoietin (TPO) levels. High cytokine activity can increase platelet use and destruction. These processes can combine and lead to thrombocytopenia during infection [4].

Persistent ITP extending for years after acute COVID‐19 remains rarely reported. Understanding such long‐term sequelae is of clinical importance, particularly for clinicians managing patients with chronic cytopenias following SARS‐CoV‐2 infection. We present a case of ITP persisting more than 4 years after COVID‐19, underscoring the need for ongoing surveillance and individualized management strategies in this patient population.

### 1.1 Case History/Examination

We present a case of a 52‐year‐old man with no prior hematologic or autoimmune disease who was diagnosed with COVID‐19 in June 2020 by reverse transcription polymerase chain reaction (RT‐PCR). His illness was moderate, managed conservatively at home with symptomatic treatment. He did not require hospitalization, oxygen therapy, or intravenous corticosteroids during the acute phase.

Following recovery, the patient had persistent fatigue, low energy, and poor appetite. Routine laboratory evaluation during his first post‐COVID follow‐up revealed isolated thrombocytopenia with a platelet count of 60,000/mm^3^. Hemoglobin and white blood cell counts were within normal limits. Liver and renal function tests (RFTs) were unremarkable. Secondary causes of thrombocytopenia were also excluded. Serologic testing for hepatitis B surface antigen, hepatitis C antibody, and HIV was insignificant. Ultrasound abdomen revealed no organomegaly. Additional investigations, including blood malarial parasite (MP), glucose, erythrocyte sedimentation rate (ESR), serum total lipids, vitamin B12, folate, cholesterol, and triglycerides were also normal. All the coagulation parameters, including PT, APTT, INR, and BT, were checked and remain normal. Additional autoimmune antibody panels were not pursued due to the absence of clinical features suggestive of systemic autoimmune disease. Stool antigen for Helicobacter pylori was done and remained negative. There was no history of recent drug use, alcohol consumption, or systemic illness. Antinuclear antibody and thyroid function tests were normal. Longitudinal laboratory evaluation, including a complete blood count (CBC), coagulation profile (PT, aPTT, INR), lipid profile, RFTs, and liver function tests (LFTs) demonstrated stable values over time. However, platelet count over the years is shown in Table 1. Peripheral blood smear showed reduced platelets with giant morphology and no abnormal cells as shown in Figure 1.

**TABLE 1:** The table shows platelet count over the years.

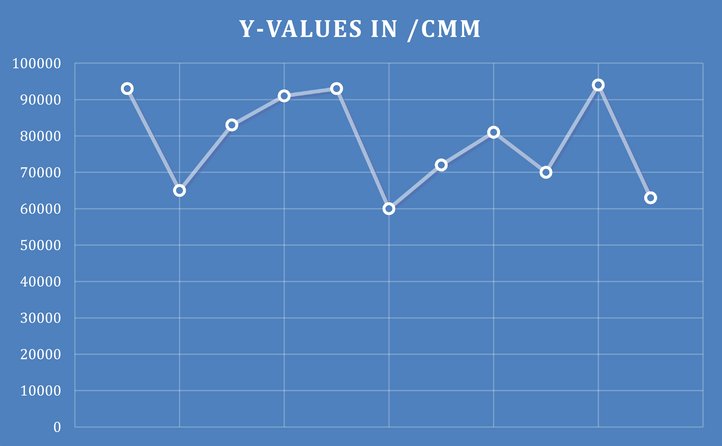

**FIGURE 1:**
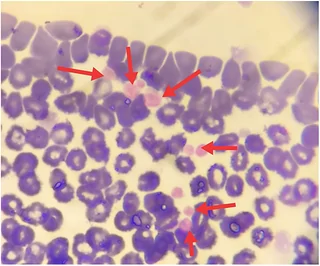
Peripheral smear showing giant platelets.

There were no subsequent COVID‐19 infections recorded, and the patient is up to date on all vaccinations till date. Additionally, the patient has no past or current comorbidities, and the past surgical history is notable only for a deviated nasal septum correction 35 years ago.

### 1.2 Differential Diagnosis and Treatment

A provisional diagnosis of ITP was established. The patient was started on a tapering course of oral corticosteroids for 1 month, resulting in an initial rise in platelet count to 94,000/mm^3^. However, the platelet counts gradually declined after the therapy was discontinued. A bone marrow biopsy was performed, revealing that cellular marrow with normal megakaryocytes, erythropoiesis, and myelopoiesis were active. Lymphopoiesis was prominent, plasma cells were within normal range, and no extramedullary cells were identified. There was no dysplasia or infiltration, confirming the diagnosis of ITP. Autoimmune serology beyond ANA was not pursued due to the absence of systemic features suggestive of connective tissue disease. Clonality studies were not performed because there were no morphological features suggestive of lymphoproliferative disease.

Over the next 5 years, the patient's platelet count remained between 60,000 and 94,000/mm^3^ on serial monitoring as shown in Table 1. Evans syndrome was considered unlikely because hemoglobin levels, reticulocyte count, bilirubin, and lactate dehydrogenase remained normal throughout follow‐up. The patient did not experience bleeding episodes, petechiae, or ecchymoses. Management remained conservative with regular follow‐up every 6 months, avoidance of NSAIDs and the patient was counseled regarding bleeding risk. The persistence of thrombocytopenia beyond 4 years after acute COVID‐19 established a diagnosis of chronic post‐COVID ITP.

## 2 Discussion

Our case highlights a significant, yet still emerging a long‐term consequence of COVID‐19 persistent immune thrombocytopenic purpura. ITP is essentially a condition where the body's own immune system mistakenly attacks and destroys its platelets, which leads to a persistently low platelet count, thereby increasing the risk of bleeding [5]. Clinical presentation varies widely; some individuals remain asymptomatic, whereas others develop bruising, epistaxis, bleeding gums, or petechiae. In rare but serious instances, this can escalate to life‐threatening hemorrhage, such as intracranial or intra‐abdominal bleeding, demanding immediate medical intervention. A diagnosis of ITP is typically made when the platelet count falls below 100,000/mm^3^. It is particularly concerning when counts drop below 20,000/mm^3^, as this significantly increases the likelihood of serious bleeding episodes [6].

What distinguishes this case from the majority of previously reported post‐COVID ITP cases is the duration and stability of thrombocytopenia. Most published reports describe ITP developing during or shortly after acute COVID‐19 infection, with resolution or partial response within weeks to months. In contrast, our patient demonstrated persistent thrombocytopenia with platelet counts remaining between 60,000 and 94,000/mm^3^ over a 5‐year follow‐up period, with no hemorrhagic complications and no requirement for escalated immunosuppressive therapy. This sustained, stable pattern of immune‐mediated platelet suppression suggests that SARS‐CoV‐2 may trigger a qualitatively distinct and longer lasting form of immune dysregulation than that seen with other viral triggers.

However, severe bleeding in ITP is uncommon, but when it occurs, it can be life‐threatening. Bleeding in the brain, gut, urinary tract, or heavy menstrual bleeding are the key reasons to start treatment in ITP. The risk of serious bleeding rises sharply when platelet counts drop below 20,000/mm^3^, in both children and adults. Most patients with ITP present with mild symptoms such as bruising or petechiae [7]. Some may have mucosal bleeding, including bleeding gums or nosebleeds, whereas in more severe cases, there may be bleeding from the gastrointestinal or urinary tract, seen as blood in the stool or urine, or heavy menstrual bleeding [8].

We report a case of a 52‐year‐old male patient, diagnosed with post‐COVID ITP. From case presentation and investigations, the case is proven to be primarily attributed to COVID‐19 with persistent ITP for the last 5 years. Several reports have described ITP during or shortly after acute COVID‐19, but long‐term persistence beyond 1 year is rarely documented. Viral infections can trigger immune reactions that target platelets. Some viral proteins resemble platelet antigens. Our immune system may form antibodies against the virus that also attach to platelets. This cross‐reactivity can increase platelet destruction and contribute to ITP during viral illnesses. One of the many viruses associated with this is the COVID‐19 [9]. ITP has been linked to various viral infections such as hepatitis C, HIV, CMV and influenza, rubella, mumps, varicella, parvovirus, and Epstein–Barr virus [10]. However, the association with SARS‐CoV‐2 is now increasingly recognized, though cases of ITP persisting beyond 1 year after acute infection remain scarcely reported in the literature. Infectious etiologies of ITP are felt to adapt molecular mimicry. This is a process whereby viral antigen is recognized as being similar to a platelet antigen, giving rise to cross‐reactive anti‐platelet autoantibodies [11].

Corticosteroids, IVIG, splenectomy, and anti‐RhD immunoglobulin have been long‐standing options for managing ITP, but these treatments often suppress the immune system and create risks during prolonged use. In recent years, many clinicians have moved toward therapies that avoid immune suppression. In recent years, TPO receptor agonists have gained prominence as preferred agents, as they augment platelet production without broadly compromising immune function. This change became more important during the COVID‐19 pandemic, when patients on immunosuppressive drugs faced a higher chance of severe infection. The pandemic highlighted the need for safer treatment strategies that protect both platelet levels and immune function. Many experts encouraged the use of options that do not interfere with antiviral defense. TPO receptor agonists gained more attention because of their safety profile in this setting. They offer reliable platelet support with fewer systemic effects. This shift in practice has continued even after the early stages of the pandemic. Clinicians now consider non‐immunosuppressive treatments an essential part of modern ITP management [12].

In September 2021, Dr Tomás José González López and Spanish ITP Group advised clinicians to avoid immunosuppressive drugs during the COVID‐19 pandemic. Their main concern was the higher risk of severe SARS‐CoV‐2 infection in patients receiving medications like rituximab. These treatments weaken immune defenses and may reduce the ability to clear viral infections. The group emphasized that patients with ITP who already face added health risks using strong immunosuppressive therapy could place them in greater danger during periods of widespread viral transmission. This guidance encouraged doctors to consider safer alternatives whenever possible. Many clinicians shifted their approach and relied more on treatments that do not lower immunity. The recommendation also influenced long‐term practice patterns and strengthened the move toward non immunosuppressive strategies for ITP management [13].

Treatment decisions in ITP often depend on patient age and clinical features, and therapies like corticosteroids and IVIG remain effective options for many children because they respond quickly and tolerate these drugs well. Older adults experience more side effects with the same medications, which pushes clinicians to consider alternatives that offer better safety in this age group. In our patient, the decision to initiate corticosteroids at a platelet count of 60,000/mm^3^ was driven by the presence of persistent constitutional symptoms and limited local access to specialist hematology services at the time of presentation. The partial response to corticosteroids is acknowledged. Long‐term management with observation was chosen because the platelet count remained stable above bleeding risk thresholds and the patient had no hemorrhagic manifestations. Advanced immunologic testing, such as platelet associated antibodies, T cell subset analysis, and cytokine profiling, was not available.

From a public health perspective, this case draws attention to the potential burden of chronic hematologic sequelae in post‐COVID populations. As millions of patients worldwide have recovered from SARS‐CoV‐2 infection, primary care physicians, general practitioners, and internists must maintain the awareness of post‐COVID immune dysregulations, including ITP, as a cause of unexplained or persistent thrombocytopenia and should initiate a diagnostic workup rather than attributing low platelet counts to incidental or benign causes. Systemic hematologic follow‐up in recovered COVID‐19 patients, particularly those with constitutional symptoms, may facilitate earlier diagnosis and prevent adverse outcomes.

## 3 Conclusion

This case demonstrates that ITP can persist for several years after recovery from COVID‐19. The persistence of thrombocytopenia in this patient suggests that SARS‐CoV‐2 may trigger long‐lasting immune dysregulation rather than a transient post‐infectious response, and that this may represent a distinct phenotype within the spectrum of post‐COVID hematologic sequelae. Clinicians should consider post‐COVID ITP in patients presenting with unexplained or chronic low platelet counts. Exclusion of secondary causes, confirmation through bone marrow evaluation, and individualized treatment planning are essential. Continuous follow‐up is required to monitor platelet trends and prevent bleeding complications. Early recognition and continuous monitoring can improve outcomes and guide future understanding of post‐COVID hematologic sequelae.

## Author Contributions

Conceptualization, writing – original draft preparation, writing – review and editing, patient management and clinical follow‐up, supervision of case report preparation, and final approval of manuscript were done by Riazullah, **Aafeen Mujeeb**, **Hakim Ullah Wazir**, **Hameed Ur Rehman**, **Hamid Ahmad**, and **Inibehe Ime Okon**.

## Funding

The authors have nothing to report.

## Ethics Statement

The need for ethical approval was waived by the Medical Research Circle Ethics Committee. The Medical Research Circle (MedReC) in Bakavu, Democratic Republic of the Congo, designated IRB has granted an exemption for clinical case studies. Ethical approval was not required for this study in accordance with local and national guidelines. All procedures performed were conducted in accordance with the ethical standards of the institutional and/or national research committee and with the 1964 Declaration of Helsinki and its later amendments.

## Consent

Informed consent was obtained from all the individual participants for participation in this study. The authors affirm that valid informed consent was given by the patient for publication of this case, including the use of clinical images, prior to manuscript submission.

## Conflicts of Interest

The authors declare no conflicts of interest.

## Patient Perspective

The patient expressed relief after finally understanding the cause of his persistent low platelet count following COVID‐19 infection. He was anxious about the possibility of a serious underlying disease but felt reassured after diagnosis and treatment. He appreciated the regular follow‐up and monitoring, which helped him understand and manage his condition and avoid complications.

## Data Availability

All data generated or analyzed during this study are included in this article. Further enquiries can be directed to the corresponding author.
